# Inter-reader agreement of RECIST and mRECIST criteria for assessing response to transarterial chemoembolization in hepatocellular carcinoma

**DOI:** 10.1186/s12880-025-01688-z

**Published:** 2025-05-03

**Authors:** Saeed Mohammadzadeh, Alisa Mohebbi, Ali Abdi, Afshin Mohammadi

**Affiliations:** 1 Universal Scientific Education and Research Network (USERN), Tehran, Iran; 2 School of Medicine, Tehran University of Medical Sciences, Tehran, Iran; 3 Department of Radiology, Faculty of Medicine, Urmia University of Medical Science, Urmia, Iran

**Keywords:** RECIST, mRECIST, Computed tomography (CT), Hepatocellular carcinoma (HCC), Transarterial chemoembolization (TACE)

## Abstract

**Objectives:**

To evaluate the reproducibilities of Response Evaluation Criteria in Solid Tumors (RECIST) and modified RECIST (mRECIST) for hepatocellular carcinoma (HCC) patients treated with transarterial chemoembolization (TACE) using contrast-enhanced computed tomography (CT).

**Methods:**

This retrospective study included 105 consecutive patients with confirmed HCC recruited from November 2002 to June 2012. The study protocol has been pre-registered at (https://osf.io/nxg4q/) on the Open Science Framework (OSF) platform. Patients with pre-procedural and follow-up CT scans who had solely received TACE were included. The tumor response evaluation to TACE was conducted using RECIST 1.1 and mRECIST guidelines. Three experienced board-certified abdominal radiologists interpreted CT scans.

**Results:**

For pre-procedure CT, the agreement was more excellent when using RECIST guidelines with a “marginally significant” p-value of 0.056. This trend continued for post-procedural CT scans, with RECIST again showing significantly higher agreement with a p-value of 0.001. When evaluating the four categories of response, Gwet’s coefficient was 0.90 (CI = 0.83 to 0.97) for RECIST and 0.80 (CI = 0.63 to 0.90) for mRECIST. Conversely, the Fleiss Kappa analysis demonstrated a higher agreement for the mRECIST guideline. There was an insignificant difference in RECIST and mRECIST guidelines inter-reader agreement when categorizing the tumor response with a p-value of 0.101.

**Conclusion:**

Both guidelines’ inter-reader reproducibility in assessing tumor response through CT after the TACE procedure was excellent, with RECIST’s reproducibility being very slightly better.

**Supplementary Information:**

The online version contains supplementary material available at 10.1186/s12880-025-01688-z.

## Introduction

Hepatocellular carcinoma (HCC) is the most common type of liver tumor and ranks fourth in terms of causing cancer-related death globally [1, 2]. The mortality rate associated with HCC in the United States has increased from 3.48 per 100,000 individuals in 2007 to 4.41 per 100,000 in 2016. The high occurrence of chronic viral hepatitis and nonalcoholic fatty liver disease likely contributes to this increase [3]. Despite advancements in HCC treatment, the prognosis remains poor due to limited access to curative alternatives such as orthotopic liver transplantation, surgical resection, and local ablative therapy, which are only available to a small subset of patients with early-stage HCC. However, transcatheter arterial chemoembolization (TACE) has shown to be a safe treatment modality for unresectable HCC patients [4]. TACE is the primary treatment for patients with advanced-stage HCC and substantially decreased liver function whose prognosis was already poor at the time of diagnosis [5].

The assessment of treatment efficacy following TACE often relies on radiological imaging techniques such as magnetic resonance imaging (MRI) or computed tomography (CT) [6]. Precise tumor response assessment is crucial for predicting patient prognosis and determining further treatment approaches [7]. The Response Evaluation Criteria in Solid Tumors (RECIST) is the established approach for assessing the effectiveness of treatment in solid tumors. As a measure based on tumor size, RECIST does not consider tumor necrosis following treatment. Consequently, The American Association for the Study of Liver Diseases (AASLD) suggested integrating changes in tumor enhancement into a modified version of the RECIST, known as mRECIST [8, 9]. Tumor viability, as indicated by the presence of contrast enhancement during the arterial phase of imaging, forms the foundation of mRECIST. The mRECIST prioritizes reducing the tumor load over simply decreasing the tumor size.

Multiple studies have evaluated the percentage change in tumor enhancement visually, a method frequently employed in clinical practice [10, 11]. Enhancing tissue can be assessed through various methods, including volumetric approaches [12], one-dimensional or two-dimensional measurements [13], or a combination of these techniques. Furthermore, in all of these studies, the magnitude of the response was evaluated without considering inter-reader agreement. In clinical practice, images are analyzed by radiologists who possess differing levels of expertise. Evaluating reader consensus is essential for quantitative assessments, particularly regarding their influence on patient care [14]. This study investigated the agreement among various readers in utilizing RECIST and mRECIST guidelines to assess tumor response in patients with HCC following TACE therapy.

## Materials and methods

The study protocol has been pre-registered at (https://osf.io/nxg4q/) on the Open Science Framework (OSF) platform. (see Appendix A). The Handbook of Inter-Rater Reliability [15, 16] has served as the foundation for the methodology and analysis. All participants provided the written informed consent, and local institutional review board approved the study. Participants were retrospectively enrolled from the HCC-TACE-Seg dataset gathered at the University of Texas, MD Anderson Cancer Center [17].

### Patient selection

The study included 105 consecutive adult patients with confirmed HCC by histopathological evaluation, recruited from November 2002 to June 2012. Pre-TACE biopsy examinations in patients identified 34 HCC tumors classified as stage I, 23 as stage II, 30 as stage III, and 18 as stage IV. HCC tumor differentiation in pathological evaluation included 37 well, 31 moderate, 1 well-to-moderate, 1 moderate-to-poor, and 13 poor differentiations, with 22 cases not stated. The study included HCC patients who met the following criteria: [1] received TACE as their first-line therapy or initial bridging therapy [2], had multi-phasic contrast-enhanced CT images with liver protocol taken before the TACE procedure (i.e., pre-procedural or baseline scans) [3], had multi-phasic contrast-enhanced CT images with liver protocol taken within 14 weeks after the TACE procedure (i.e., post-procedural or follow-up scans), and [4] CT images were of good quality without any noticeable artifacts. Pre-procedural CT images were acquired 1–12 weeks before the first TACE session with an average 3-week interval. Both typical and atypical tumors according to arterial wash-in and venous wash-out imaging were included to enhance generalizability. As tumor size of up to 5 cm and single nodularity were indicative of a full response to TACE, while multinodularity (multiple nodules within the same lesion) and higher tumor size correlated with recurrence [18], we included 52 single nodular and 53 multinodular HCC lesions to ensure generalizability. Patients undergoing TACE with multiple HCC lesions were not included in the study to ensure that the computation of Overall Survival (OS) and Time to Progression (TTP) is based only on one lesion and is not influenced by any other confounder. Furthermore, tumor characteristics such as vascular invasion, lymph node involvement, distant metastasis, and portal vein thrombosis were also collected. Drug-eluting bead TACE (DEB-TACE) (40 patients) and cTACE (65 patients) were the two types of TACE procedures utilized in our study. Patients undergoing TACE received one of the following chemotherapy protocols: (a) doxorubicin loaded in 20- to 100-mg drug-eluting beads (LC Beads, DEBDOX, BTG International, London, England) or (b) a combination of cisplatin (100 mg), doxorubicin (50 mg), and mitomycin C (10 mg).

### Image acquisition

All patients had contrast-enhanced CT scans of the abdomen using either 16- or 64-detector row CT scanners (LightSpeed; GE Healthcare, Waukesha, WI, USA) with a liver protocol. A pre-contrast scan was acquired, followed by an arterial phase scan 17 s after the aorta reached the peak enhancement, utilizing bolus tracking to monitor the infusion of contrast media. The porto-venous phase was detected at 60 s and delayed phase was detected at 150 s. A total of 621 CT series (pre-procedural and post-procedural multi-phasic scans) from 105 individuals were analyzed.The images were obtained using the following scanner parameters: Tube current of 150–630 mA; CT tube voltage of 120–140 KVp; slice thickness of 0.63–5 mm; table speed of 18.75–39.38; table speed of 18.75–39.38 mm/gantry rotation; revolution time of 0.40–0.80 s; field of view of 360–460 mm; and Pitch of 0.9–0.98.

### Tumor response assessment

The evaluation of tumor response to TACE was conducted using RECIST 1.1 and mRECIST guidelines [19, 20]. All pre- and post-procedural investigations were interpreted by three board-certified radiologists, each with more than 20 years of experience in abdominal imaging. The readers who were blinded to the patient’s clinical characteristics independently assessed the size of tumors in both pre- and post-procedural tests, considering factors such as tumor viability and enhancement during the arterial phase. The tumor size change through the TACE procedure was calculated using the following formulas:

$$\:\frac{(Post-TACE\:RECIST\:-\:Pre-TACE\:RECIST)}{Pre-TACE\:RECIST}$$ ×100

$$\:\frac{(Post-TACE\:\text{m}RECIST\:-\:Pre-TACE\:\text{m}RECIST)}{Pre-TACE\:\text{m}RECIST}$$ ×100

Also, tumor response was categorized into four groups: (1) complete response (2), partial response (3), stable disease, and (4) progressive disease [17]. Complete response was defined as either the disappearance of all target lesions (RECIST) or the disappearance of any intratumoral arterial enhancement in all target lesions (mRECIST); partial response was characterized as at least a 30% decrease in the sum of diameters of target lesions, taking as reference the baseline sum (RECIST), or at least a 30% decrease in the sum of diameters of viable (enhancing) target lesions, taking as reference the baseline sum (mRECIST); stable disease was described as any case not qualifying for progressive disease by either RECIST or mRECIST; and progressive disease was defined as at least a 20% increase in the sum of the diameters of target lesions, taking as reference the smallest sum recorded since treatment started (RECIST), or at least a 20% increase in the sum of the diameters of viable (enhancing) target lesions, taking as reference the smallest sum of viable (enhancing) target lesions recorded since treatment started (mRECIST).

Data processing involved the segmentation of the tumor, encompassing both viable and necrotic regions, along with the adjacent liver tissue. Manual segmentation was performed with semi-automated segmentation methods in AMIRA software (FEI, Thermo Fisher Scientific, Hillsboro, OR, USA) by three radiology residents and subsequently evaluated by a body imaging radiologist with 20 years of experience. The porto-venous phase of pre-procedural CT imaging was utilized to facilitate lesion evaluation. Pre-procedural scans for each patient, including pre-contrast, arterial, and port-venous images, were re-sampled to the port-venous phase images to facilitate lesion evaluation. The three segmentations were verified and re-sampled using the STAPLE method to yield a single image, which accurately represents the ground true segmentation. The STAPLE algorithm utilizes various segmentations as input to produce a binary image for each voxel representing the “true” segmentation. This procedure is executed on every label. The CT studies were exported in DICOM format and subsequently converted to the Neuroimaging Informatics Technology Initiative (NIFTI) format. Three radiology residents conducted segmentation using semi-automated tools available in AMIRA software (FEI, Thermo Fisher Scientific, Hillsboro, OR, USA). The Convert3D medical image processing program, part of the ITK-SNAP software package, was utilized for all image manipulations.

### Statistical analysis

Statistical analyses were performed utilizing Stata 17 and Medcalc 22.017. We calculated the intraclass correlation coefficient (ICC) type 1 A for the evaluation of agreement among quantitative variables. The findings of type 1 A ICC are applicable from the patient sample (*n* = 105) to the broader patient population, as well as from the rater sample (*n* = 3) to the overall rater population. The agreement was categorized as poor for ICC values less than 0.50, moderate for values between 0.50 and 0.75, good for values between 0.75 and 0.90, and excellent for values equal to or greater than 0.90 [21]. Bland-Altman (BA) graphs were plotted to further assess visual agreement interpretation. The evaluation of categorical data employed Fleiss kappa and Gwet’s coefficients. The unconditional subtype was employed for the same rationale as ICC, allowing for statistical generalization of findings from the patient sample to the patient population and from the rater sample to the rater population. The agreement was classified as poor for values of 0, slight for 0-0.2, fair for 0.2–0.4, moderate for 0.4–0.6, substantial for 0.6–0.8, and perfect for values greater than 0.8. Ordinal weighting was utilized to assess agreement among categorical variables with multiple conditions. The inter-rater agreement coefficients were compared between RECIST and mRECIST criteria to draw a conclusion. A p-value below 0.05 was considered statistically significant.

## Results

### Patient characteristics

A total of 105 patients were involved in our database, with 68 male patients (average age: 66.4 years [range: 31–88]) and 37 female patients (average age: 69.6 years [range: 46–93]). Of these patients, 93 (88.6%) died, and the mean follow-up of patients was 126.6 weeks (range: 106–146). The mean tumor size was 5.63 cm (CI = 4.54 to 6.71). 82 lesions were located in the right hepatic lobe, while 23 lesions were located in the left hepatic lobe. Out of the total number of patients, 7 (6.7%) had metastasis, 14 (13.3%) had lymph node involvement, 23 (21.9%) had vascular invasion, and 16 (15.2%) had portal vein thrombosis. A summary of the tumor response assessment data of the patients in regard to each reader’s interpretation is provided in Table 1.

**Table 1:** Reader’s interpretation data in relation to tumor response

		Reader No.1	Reader No.2	Reader No.3
Mean tumor size based on pre-procedural RECIST		77.92 (CI = 68.58–87.26)	80.15 (CI = 70.36–89.94)	77.66 (CI = 67.89–87.43)
Mean tumor size based on post-procedural RECIST		74.12 (CI = 64.20-84.03)	74.62 (CI = 64.40-84.85)	72.52 (CI = 62.12–82.93)
Mean tumor size based on pre-procedural mRECIST		70.62 (CI = 62.05–79.18)	73.14 (CI = 63.83–82.45)	67.42 (CI = 58.90-75.95)
Mean tumor size based on post-procedural mRECIST		44.60 (CI = 36.14–53.06)	42.22 (CI = 33.02–51.41)	42.23 (CI = 33.73–50.73)
RECIST treatment response category	2: partial response	9 (9.38%)	10 (10.42%)	11 (11.58%)
	3: progressive disease	77 (80.21%)	80 (83.33%)	76 (80.00%)
	4: stable disease	10 (10.42%)	6 (6.25%)	8 (8.42%)
mRECIST treatment response category	1: complete response	21 (21.88%)	26 (27.08%)	14 (14.74%)
	2: partial response	40 (41.67%)	41 (42.71%)	45 (47.36%)
	3: progressive disease	29 (30.21%)	25 (26.04%)	32 (33.68%)
	4: stable disease	6 (6.25%)	4 (4.17%)	4 (4.21%)

### Pre- and post-procedural agreement

**Fig. 1:**
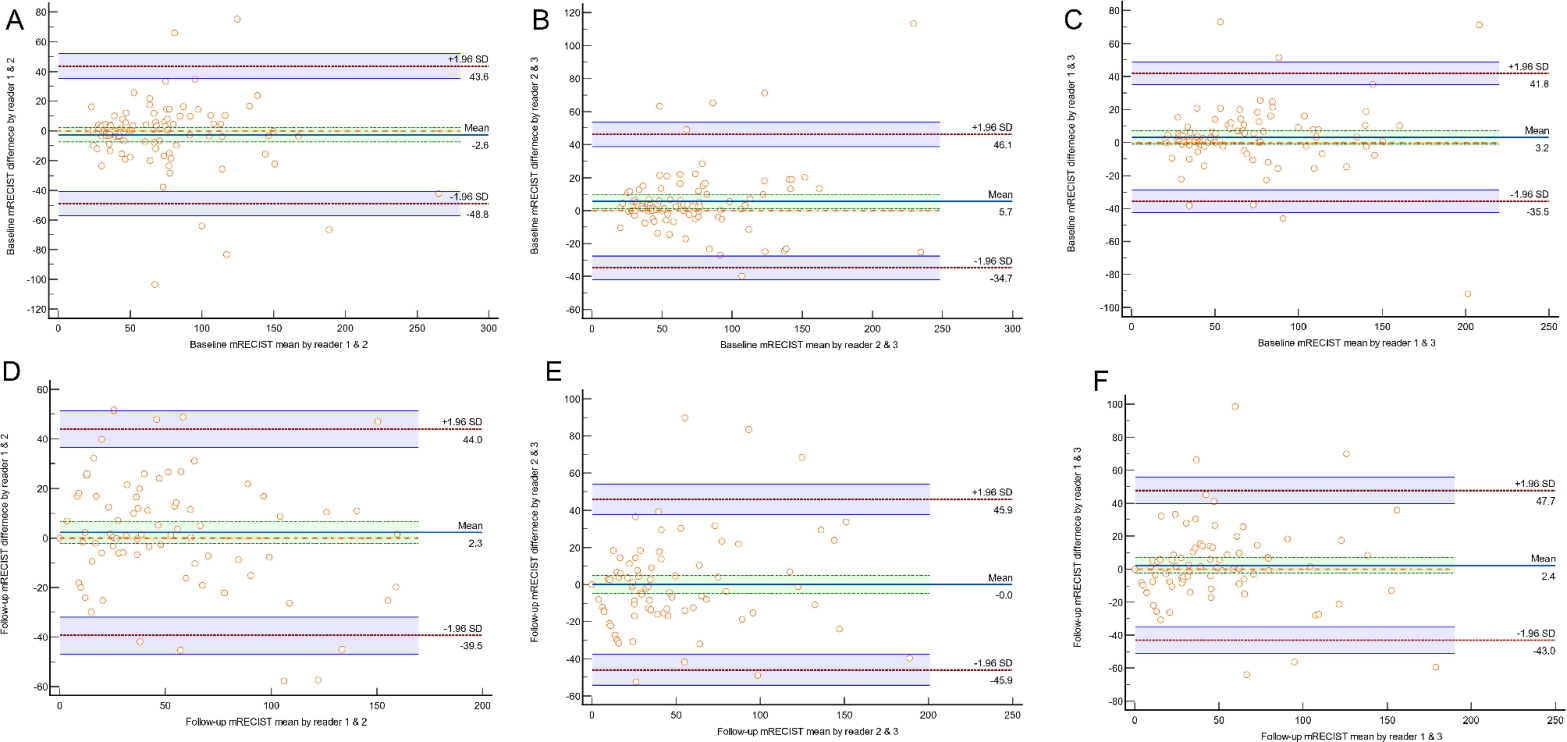
(**A**) Pre-procedure mRECIST Bland-Altman (BA) plot of reader 1&2. (**B**) Pre-procedure mRECIST BA plot of reader 2&3. (**C**) Pre-procedure mRECIST BA plot of reader 1&3. (**D**) Post-procedure mRECIST BA plot of reader 1&2. (**E**) Post-procedure mRECIST BA plot of reader 2&3. (**F**) Post-procedure mRECIST BA plot of reader 1&3

Inter-radiologist agreement for tumor measurement was assessed using ICC. The pre-procedure RECIST and mRECIST ICCs were 0.92 (CI = 0.90 to 0.94) and 0.87 (CI = 0.83 to 0.91). The agreement was more excellent when using RECIST guidelines with a “marginally significant” p-value of 0.056. Moreover, the post-procedure RECIST and mRECIST agreement were evaluated by ICCs of 0.94 (CI = 0.92 to 0.96) and 0.86 (CI = 0.82 to 0.90). The post-procedural agreement was also more excellent when using the RECIST guideline with a significant p-value of 0.001. Bland-Altman (BA) plots for mRECIST pre- and post-procedural tests between different radiologists are depicted in Fig. 1. Also, Appendix B displays BA plots for RECIST pre- and post-procedural tests between different readers.

### Category of tumor response

As for the four categories of response, the Fleiss Kappa for the RECIST tumor response category was 0.51 (CI = 0.29 to 0.72), and Gwet’s coefficient was 0.90 (CI = 0.83 to 0.97). Also, the Fleiss Kappa for the mRECIST tumor response category was 0.60 (CI = 0.42 to 0.77), and Gwet’s coefficient was 0.80 (CI = 0.69 to 0.90). There was an insignificant superiority of RECIST over mRECIST inter-reader agreement when determining the category of response with a p-value of 0.101 based on Gwet’s coefficient. Table 2 shows different agreement measures pertaining to the inter-reader reproducibility of both guidelines. Also, the pair-wise comparison of readers in categorizing tumor response is shown in Tables 3 and 4.

**Table 2:** Agreement measures in regard to pre- and post-procedural tests as well as the category of tumor response

	Intra-class correlation coefficient (ICC)	Kappa	
		Gwet’s agreement coefficient	Fleiss agreement coefficient
Pre-procedural RECIST tumor size	0.92 (CI = 0.90 to 0.94)	N/A	N/A
Pre-procedural mRECIST tumor size	0.87 (CI = 0.83 to 0.91)	N/A	N/A
Post-procedural RECIST tumor size	0.94 (CI = 0.92 to 0.96)	N/A	N/A
Post-procedural mRECIST tumor size	0.86 (CI = 0.82 to 0.90)	N/A	N/A
RECIST treatment response category	N/A	0.90 (CI = 0.83 to 0.97)	0.51 (CI = 0.29 to 0.72)
mRECIST treatment response category	N/A	0.80 (CI = 0.69 to 0.90)	0.60 (CI = 0.42 to 0.77)

**Table 3:** Pairwise comparison of readers in categorizing tumor response based on RECIST guideline

	Reader 3 RECIST				Reader 2 RECIST				Reader 3 RECIST		
Reader 1 RECIST	(2)	(3)	(4)	Reader 1 RECIST	(2)	(3)	(4)	Reader 2 RECIST	(2)	(3)	(4)
(2)	3	5	0	(2)	5	4	0	(2)	4	4	0
(3)	7	67	2	(3)	4	69	3	(3)	7	70	3
(4)	1	4	4	(4)	0	7	3	(4)	0	2	3

(2) partial response, (3) stable disease, and (4) progressive disease

**Table 4:** Pairwise comparison of readers in categorizing tumor response based on mRECIST guideline

	Reader 3 mRECIST					Reader 2 mRECIST					Reader 3 mRECIST			
Reader 1 mRECIST	(1)	(2)	(3)	(4)	Reader 1 mRECIST	(1)	(2)	(3)	(4)	Reader 2 mRECIST	(1)	(2)	(3)	(4)
(1)	13	6	1	0	(1)	17	2	2	0	(1)	14	8	2	1
(2)	0	29	11	0	(2)	6	24	10	0	(2)	0	29	11	0
(3)	1	9	17	1	(3)	3	13	11	1	(3)	0	8	17	0
(4)	0	1	2	2	(4)	0	1	2	3	(4)	0	0	1	2

(1) complete response, (2) partial response, (3) stable disease, and (4) progressive disease

## Discussion

Over the last ten years, the mRECIST criteria have been widely employed in clinical research to evaluate tumor response, progression-free survival, and time-to-progression [22]. In patients with HCC, TACE techniques can induce necrosis prior to any reduction in tumor size. Consequently, necrosis- and enhancement-based criteria, such as mRECIST, may provide earlier predictions of outcomes compared to size-based criteria like RECIST [23]. Choi et al. [24] demonstrated that experienced radiologists exhibited a high degree of intra-reader reproducibility. Inexperienced individuals exhibited a lower level of agreement, suggesting that the performance of the mRECIST criteria is contingent upon experience. The subgroup attempted to enhance agreement by conducting a brief, 30-minute lecture on mRECIST guidelines; however, this intervention proved inadequate for ensuring consistent tumor measurement reporting among less experienced radiologists. The inter-reader agreement of mRECIST among radiologists with differing experience levels exhibits a consistent degree of uncertainty, indicating that prolonged training is essential for the proper implementation of mRECIST guidelines.

A meta-analysis by Kudo et al. [25], indicates that objective response assessed by mRECIST to systemic therapies in advanced HCC serves as an independent predictor of overall survival. These data complement similar observations for intermediate HCC treated with locoregional therapies [26]. Consequently, the mRECIST may identify those HCC patients who are more likely to derive long-term benefits from systemic therapies based on AASLD and European Association for the Study of the Liver (EASL). Consistent image acquisition protocols and accurate interpretation by qualified radiologists are essential for the effective application of mRECIST.

Our study employed unconditional ordinal weighted kappa to assess the agreement among radiologists in classifying tumor response. The ordinal weighting encompasses the full spectrum of potential ratings rather than solely focusing on marginal agreement (i.e., all radiologists selecting the same category), thereby offering a more thorough evaluation of reader agreement. The unconditional feature enhances the applicability and generalizability of our findings to real-world clinical settings, particularly for [1] other HCC patients undergoing TACE in diverse clinical environments and [2] radiologists with differing levels of expertise. A notable strength of our study is the decision to avoid dichotomizing the response assessment category into complete and incomplete responses, thereby preventing information loss. The preservation of the complete range of response categories in the evaluation scale enabled the detection of subtle differences that a binary categorization might obscure. A semi-automated tool for the determination of regions of interest (ROIs) is utilized to reduce potential human error and variability in ROI definition. The process was guided by residents’ judgment, while the software provided consistency and minimized the risk of errors associated with manual drawing.Our findings regarding the radiologist agreement in tumor evaluation at pre-procedural and post-procedural CTs demonstrated an overall “excellent” level of interpretation magnitude for both RECIST and mRECIST guidelines. Also, we found that RECIST guidelines may lead to more consistent measurements between radiologists compared to mRECIST, particularly in post-procedural CT evaluation. The BA plots presented in Fig. 1 and Appendix B also support these findings.

**Fig. 2:**
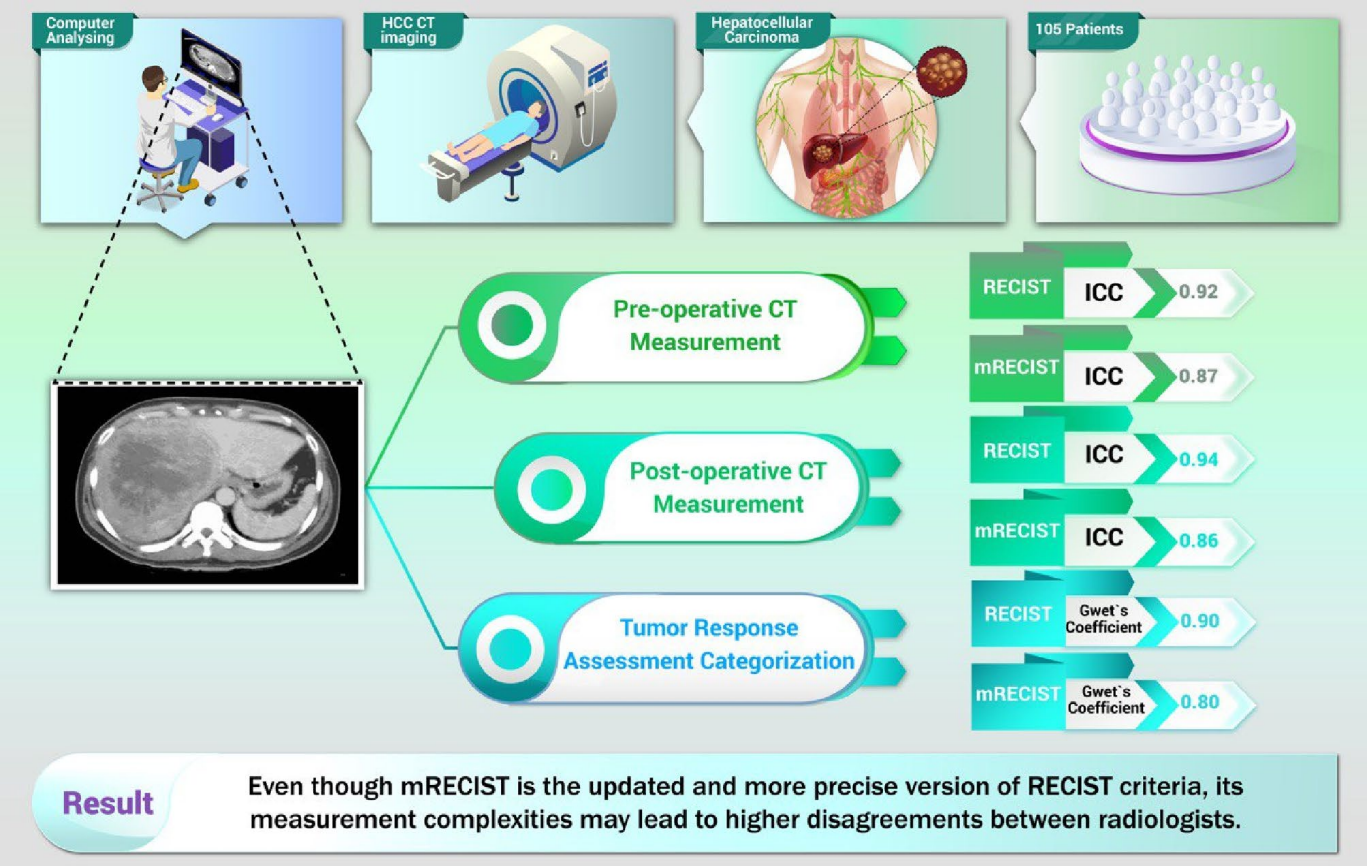
Graphical abstract containing key findings of the study

To assess tumor response, which holds greater clinical significance than individual pre- or post-procedural tests, we utilized Gwet’s coefficient to evaluate inter-reader agreement. Fleiss Kappa is a commonly utilized measure; however, Gwet’s Kappa offers greater robustness as it demonstrates reduced sensitivity to category prevalence and provides increased reliability in scenarios where agreement may occur by chance [27]. There was an “excellent” level of inter-reader agreement for interpretation magnitude for RECIST guideline and a “good” level of inter-reader agreement for interpretation magnitude for mRECIST guideline. The insignificant difference (p-value = 0.101) between Gwet’s coefficients indicated that mRECIST was non-inferior to RECIST in terms of reproducibility. Moreover, the Fleiss Kappa analysis demonstrated a higher level of inter-reader agreement for the mRECIST guideline. Our results regarding the categorization of the tumor response indicate that mRECIST should be used very cautiously for HCC response assessment as it provides a valuable guideline with more accurate performance in tumor assessment. A comprehensive graph summarizes the key findings of our study in Fig. 2.

There were some limitations in our investigation: (1) Other established guidelines such as Liver Imaging Reporting And Data System (LI-RADS) and quantitative EASL (qEASL) were not evaluated in our study. Future research could benefit from a comprehensive comparison of these additional guidelines to RECIST and mRECIST as well (2). Blinding readers to the type of guideline used was not feasible due to the subjective design of these guidelines. However, there was a time interval between pre-procedure and post-procedure evaluations, minimizing the potential for recall bias (3). The largest lesion in each patient was chosen for evaluation, and we did not assess all lesions. This approach may have introduced upward bias in our results (4). We investigated the response of HCC to TACE treatment using CT scans. While CT is a widely used imaging modality for HCC assessment, MRI can offer additional advantages [28]. Future research could focus on the potential benefits of incorporating MRI into the evaluation of HCC response to TACE (5). In order to assess the unbiased outcomes of TACE, we specifically excluded patients who had previously received any treatments associated with HCC. This was done to ensure that the outcomes of TACE were evaluated independently, without being influenced by the outcomes of any past treatment(s). This could potentially result in selection bias and limit the applicability of these findings to patients who receive TACE as a second-line therapy (6). We did not evaluate intra-reader reproducibility, which measures the consistency of measurements made by the same radiologist over time (7). We also did not evaluate the correlation between HCC lesion type (atypical vs. typical) and inter-reader agreement in TACE response assessment (8). All of our readers were experts with > 20 years of experience, which led to a potential upward bias in our reproducibility results.

## Conclusion

Evaluating the response to TACE in HCC patients is challenging, which has resulted in the development of various guidelines for radiologists. The inter-reader reproducibility of mRECIST in patients with HCC after TACE by several readers interpreting their CT scans is excellent, and the reproducibility is slightly lower than that of RECIST criteria. More experienced radiologists with a more comprehensive understanding of mRECIST may lead to an improvement in the repeatability of results.

## Electronic supplementary material

Below is the link to the electronic supplementary material.

Supplementary Material 1

Supplementary Material 2

Supplementary Material 3

## Data Availability

Open access HCC-TACE-Seg dataset on The Cancer Imaging Archive (TCIA) website https://www.cancerimagingarchive.net/collection/hcc-tace-seg/ was analyzed in this study.

## Declarations

### Animal study

N/A.

### Clinical trial number

N/A.

## Abbreviations

RECIST: Response evaluation criteria in solid tumors
mRECIST: Modified RECIST
TACE: Transarterial chemoembolization
HCC: Hepatocellular carcinoma
CT: Computed tomography
