# Supplementary material for: Inter-reader agreement of RECIST and mRECIST criteria for assessing response to transarterial chemoembolization in hepatocellular carcinoma
Source: BMC Med Imaging. 2025 May 3;25:148. doi: 10.1186/s12880-025-01688-z (PMC12049784; doi:10.1186/s12880-025-01688-z)
Supplement: Supplementary file 1 — Supplementary Material 1 [file 12880_2025_1688_MOESM1_ESM.pdf]

Comparing the agreement of radiologists in mRECIST vs. RECIST in hepatocellular carcinoma patients treated with transarterial chemoembolization

1. Review question:

To compare the agreement of radiologists in mRECIST vs. RECIST in hepatocellular carcinoma patients treated with transarterial chemoembolization.

2. Condition or domain being studied:

Hepatocellular carcinoma (HCC) is the most common primary type of liver cancer, and its occurrence has increased in the last 20 years due to rising risk factors. Although there is surveillance in place, the majority of HCC patients are detected at advanced stages and can only be managed with transarterial chemo-embolization (TACE) or systemic therapy. TACE failure can occur in up to 60% of instances, resulting in a significant financial and emotional burden for patients. TACE utilizes the differential blood supply of the liver, where HCC tumors are mainly fed by the hepatic artery and the normal liver tissue is primarily supplied by the portal vein, to administer chemotherapy specifically to the targeted liver tumors. This approach prevents the chemotherapy from causing damage to healthy liver tissue. The Response Evaluation Criteria in Solid Tumors (RECIST) guideline was introduced to assess treatment response by quantifying tumor shrinkage, which serves as an important indicator of the effectiveness of cytotoxic medications in treating tumors.

The criteria for assessing tumor responses in HCC using radiological methods have been developed in order to appropriately evaluate tumor responses. The WHO criteria and the following RECIST assess changes in tumor size. However, these criteria often overlook tumor necrosis, which might lead to an underestimation of treatment responses. Therefore, a group of specialists from the European Association for the Study of Liver (EASL) revised the criteria for evaluating treatment responses to include the consideration of tumor necrosis. In 2010, the modified RECIST (mRECIST) was created, incorporating the notions of tumor viability through arterial enhancement and single linear summation, resulting in a streamlined version of the EASL criteria. Currently, the mRECIST is considered the most popular method for radiologically assessing tumor response during HCC treatment. The objective of this retrospective study is to evaluate the agreement of radiologists in mRECIST vs. RECIST in hepatocellular carcinoma patients treated with transarterial chemoembolization.

3. Participants/population:

We will use a dataset consisting of 105 patients who were diagnosed with HCC, underwent TACE, and had CT scans of satisfactory quality without any noticeable artifacts before and

within 14 weeks after TACE. The data was collected from the institutional database of MD Anderson Cancer Center between November 2002 and June 2012. Patients with HCC and available consent were secured for this study due to open access and accessible data availability.

4. Intervention(s), exposure(s):

All patients had contrast-enhanced CT scans of the abdomen using either 16- or 64-detector row CT scanners (LightSpeed; GE Healthcare, Waukesha, WI, USA) with a liver protocol. A pre-contrast scan was acquired, followed by an arterial phase scan 17 seconds after the aorta reached its maximum enhancement, employing bolus tracking, following the administration of contrast medium. The porto-venous phase was imaged after 60 seconds. The images were obtained using the scanner parameters listed below: The CT scanner operates in a tube voltage range of 120–140 KVp, with a tube current range of 150–630 mA. The slice thickness may be adjusted between 0.63–5 mm. The pitch value is from 0.9–0.98, and the revolution duration is between 0.40–0.80 seconds. The table speed during gantry rotation is 18.75–39.38 mm per rotation, and the field of view is 360–460 mm. The contrast medium was injected at a rate of 3-5 ml per second. A conventional picture reconstruction algorithm was utilized in all instances. A total of 105 patients underwent examination of a total of 621 CT series, which included pre-procedural and post-procedural multi-phasic scans.

5. Main outcome:

To evaluate the reliability of RECIST vs. mRECIST results in HCC patients who have undergone TACE.

6. Additional outcome(s):*****

A) To calculate the baseline longest diameter of target lesion agreement (ICC) for both RECIST and mRECIST and their comparison.

B) To calculate the follow-up longest diameter of target lesion agreement (ICC) for both RECIST and mRECIST and their comparison.

C) To calculate the percentage change of the longest diameter of target lesion agreement (ICC) for both RECIST and mRECIST and their comparison.

D) To calculate the categorized response assessment agreement (kappa) for both RECIST and mRECIST and their comparison.

7. Imaging:

The pre- and post-procedural studies were evaluated by three board-certified radiologists, each with over 20 years of expertise in abdominal imaging. The researchers conducted separate

measurements of tumors in both pre- and post-procedural examinations, including factors such as tumor survivability and augmentation during the arterial phase.

8. Strategy for data synthesis:

The analysis will be conducted using STATA version 17.0 and MedCalc 22.0.

9. Contact details for further information:

Afshin Mohammadi

mohammadi.a@umsu.ac.ir

10. Team members:

Alisa Mohebbi

Saeed Mohammadzadeh

Ali Abdi

11. Type and method of research:

Original study evaluating agreement

12. Anticipated or actual start date:

June 27, 2024

13. Anticipated completion date:

July 10, 2024

14. Funding sources/sponsors:

None

15. Conflicts of interest:

None
