# Supplementary material for: Inter-reader agreement of RECIST and mRECIST criteria for assessing response to transarterial chemoembolization in hepatocellular carcinoma
Source: BMC Med Imaging. 2025 May 3;25:148. doi: 10.1186/s12880-025-01688-z (PMC12049784; doi:10.1186/s12880-025-01688-z)

Appendix B. A)Pre-procedure RECIST Bland-Altman (BA) plot of reader 1&2. B) Pre-procedure RECIST BA plot of reader 2&3. C) Pre-procedure RECIST BA plot of reader 1&3. D) Post-procedure RECIST BA plot of reader 1&2. E) Post-procedure RECIST BA plot of reader 2&3. F) Post-procedure RECIST BA plot of reader 1&3.

A)


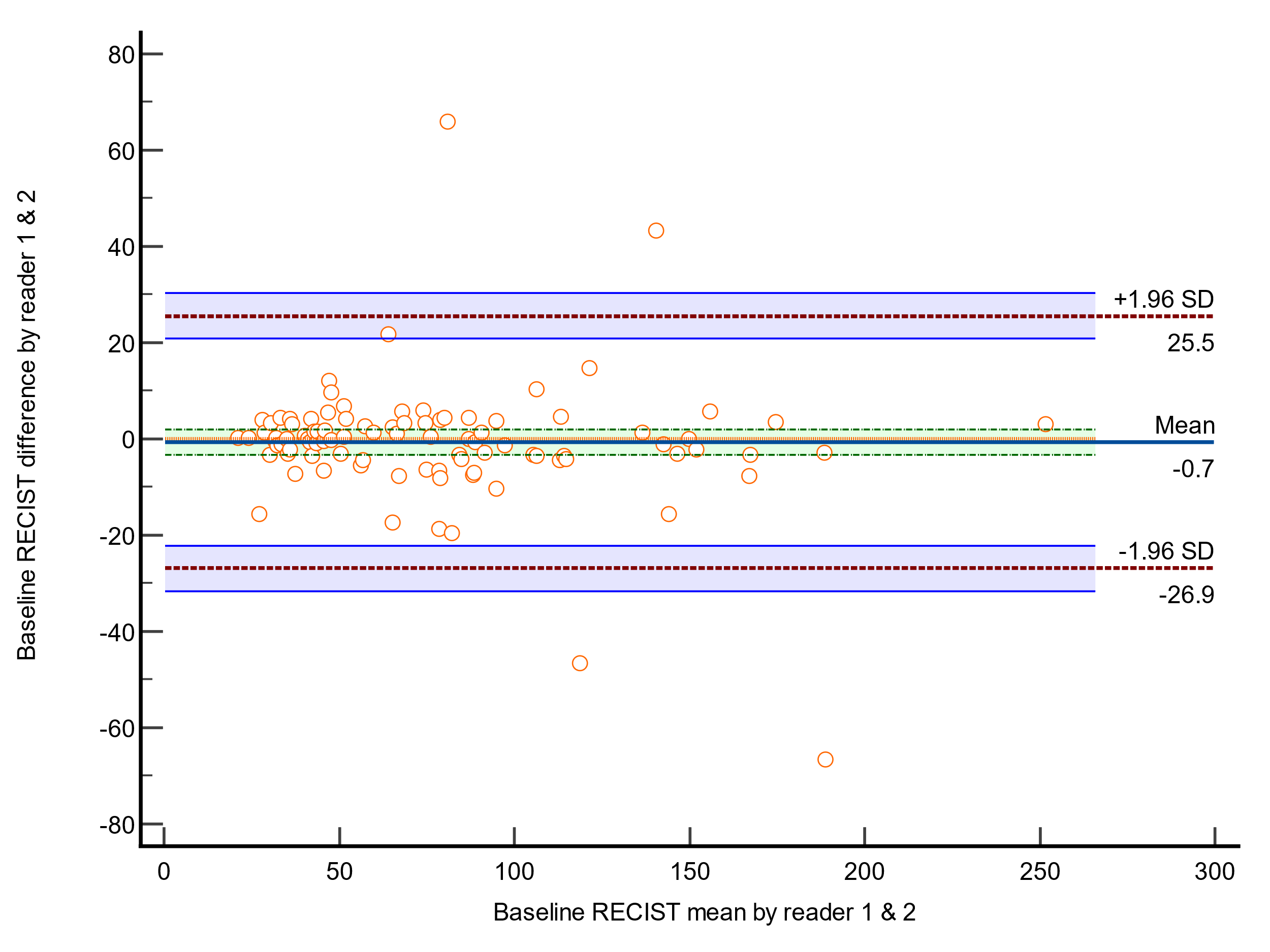


B)


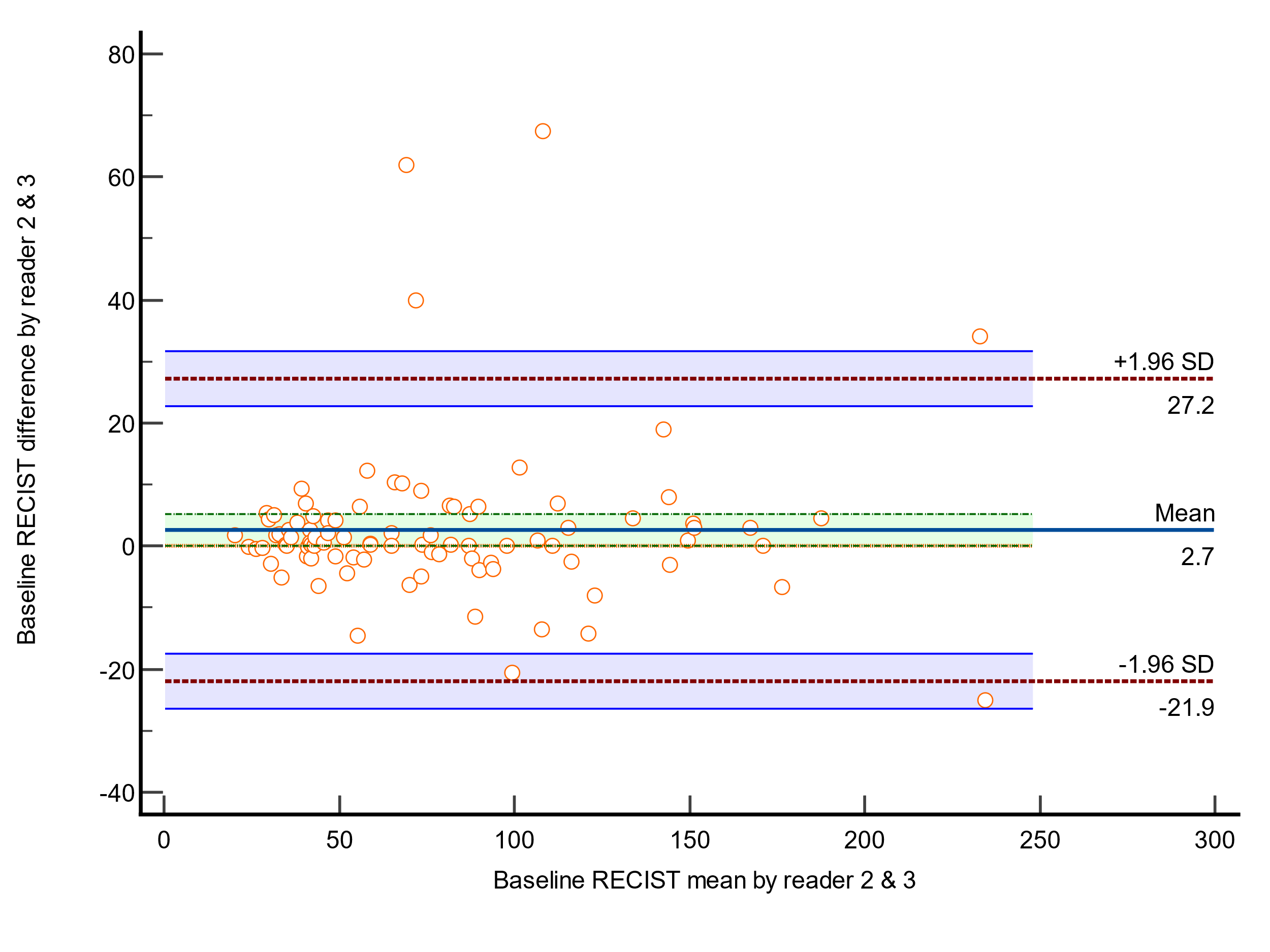


C)


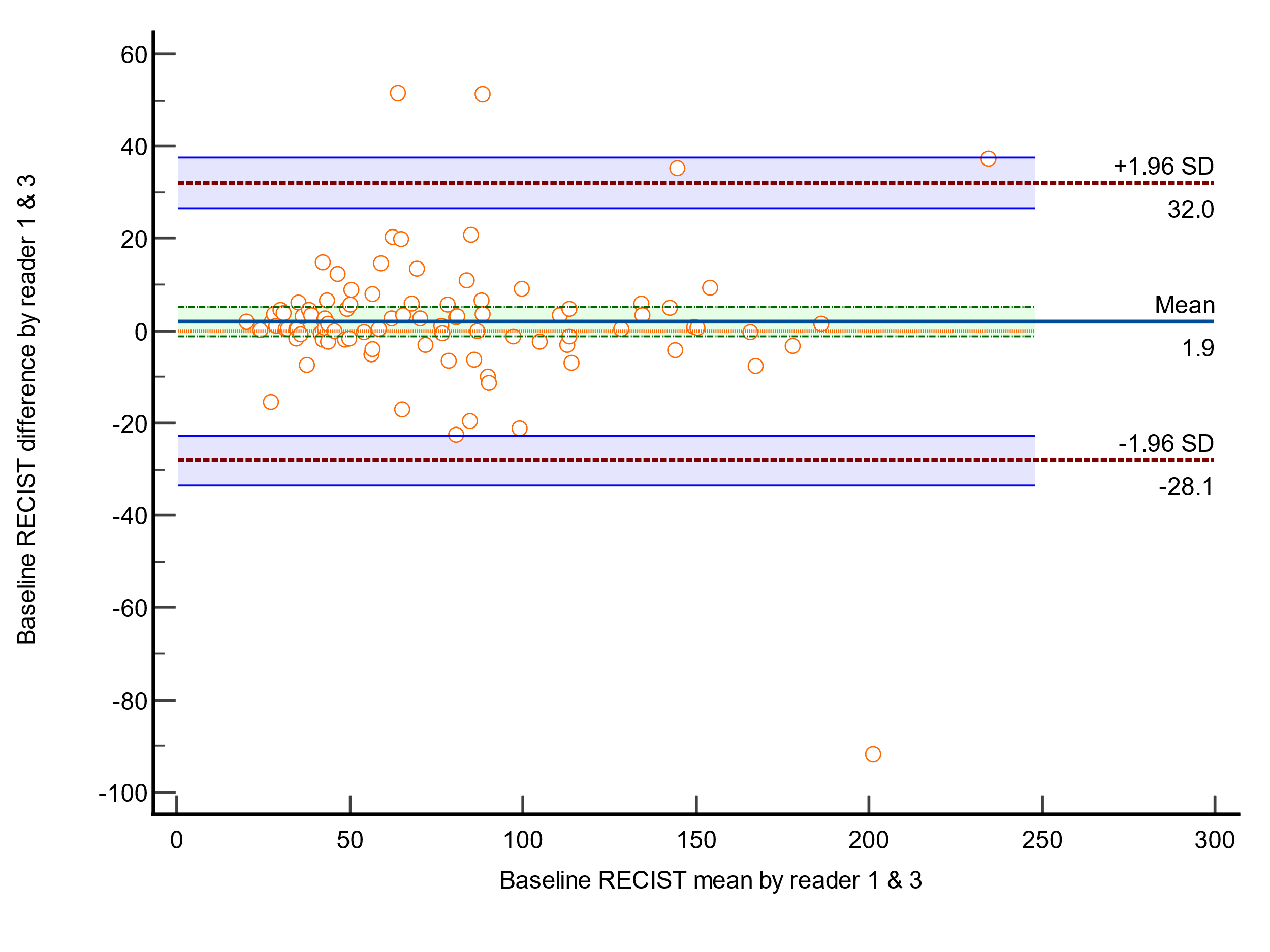


D)


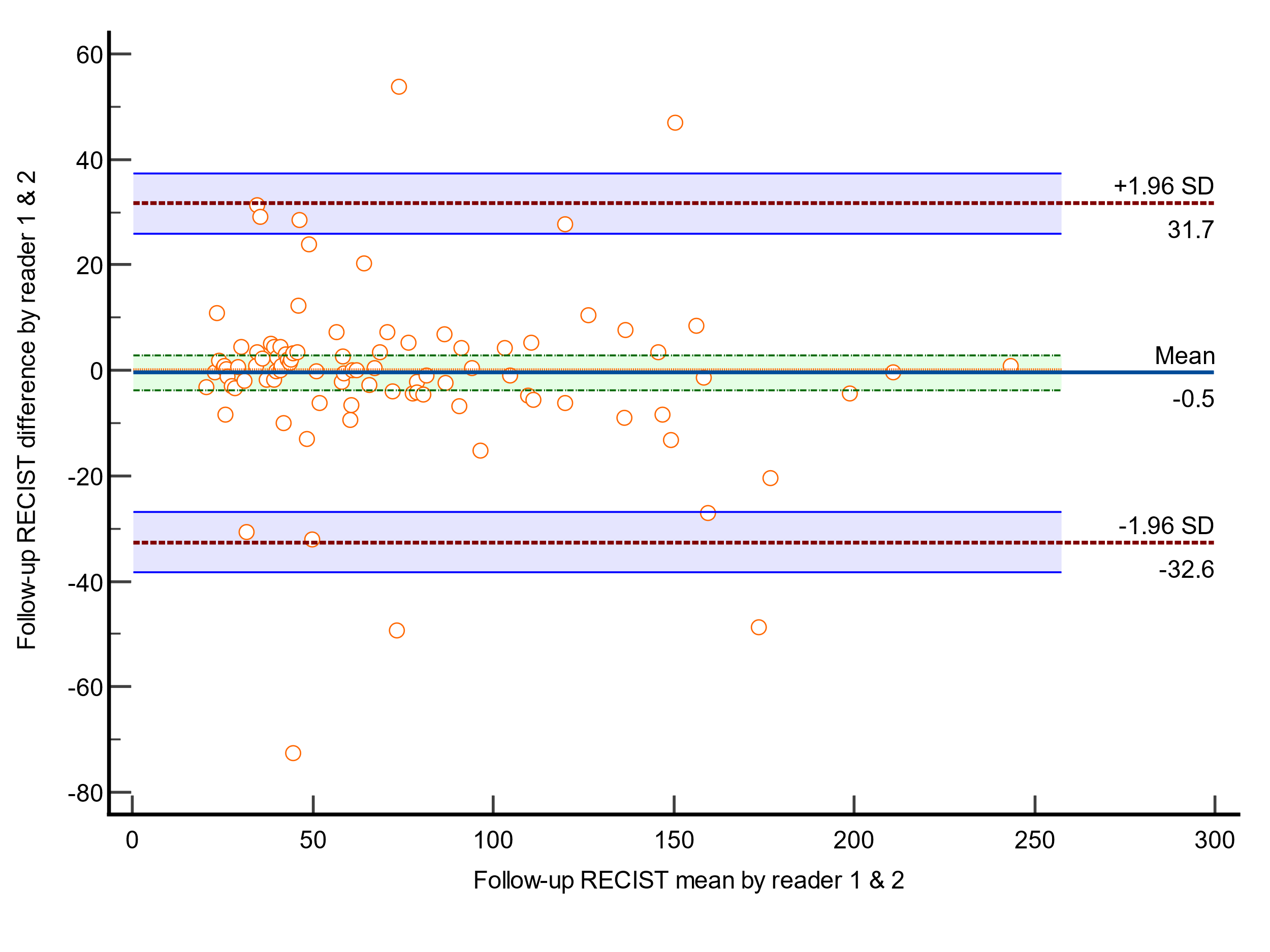


E)


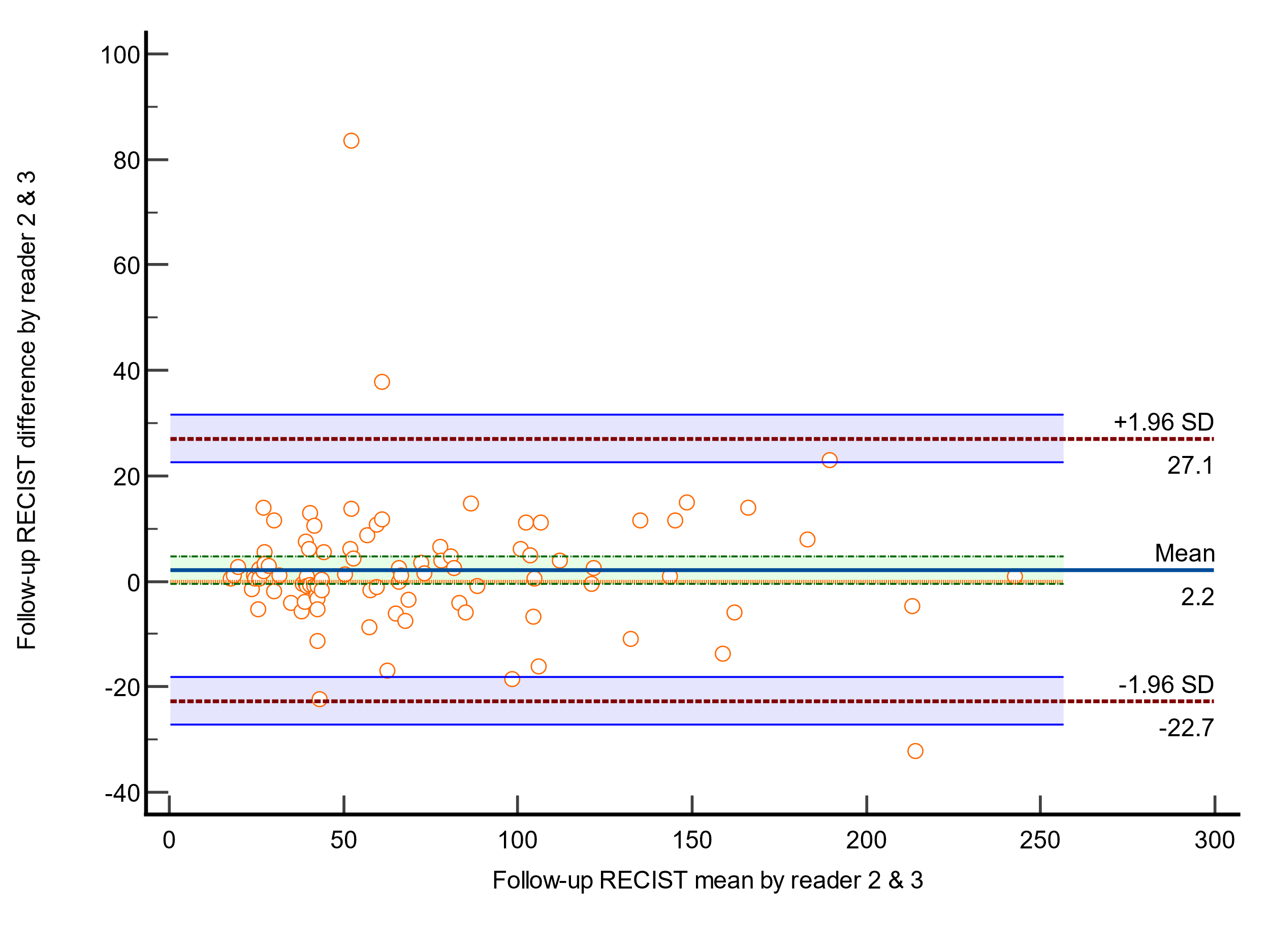


F)


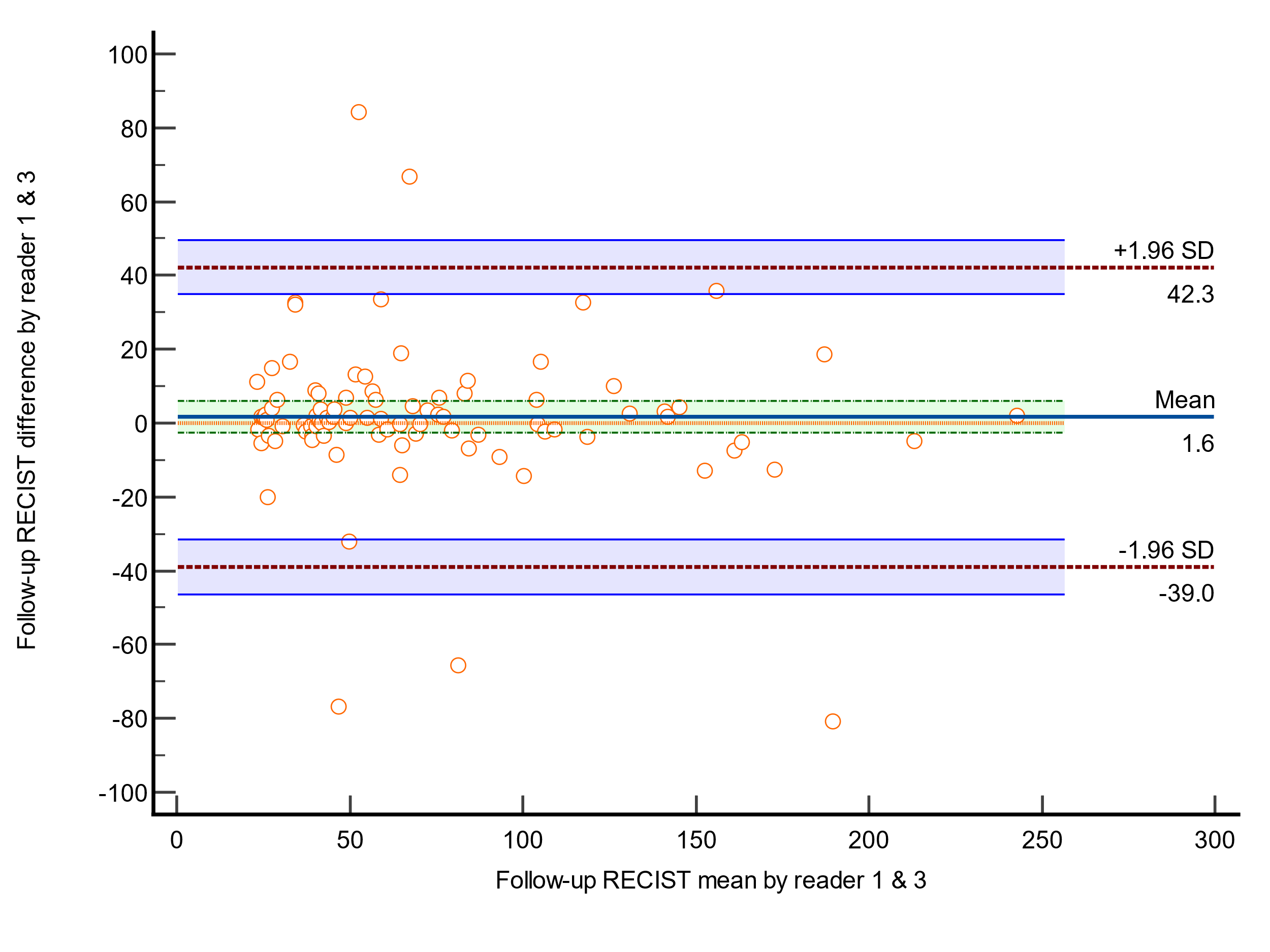

Supplement: Supplementary file 2 — Supplementary Material 2 [file 12880_2025_1688_MOESM2_ESM.docx]
